# Indirect DNA Readout by an H-NS Related Protein: Structure of the DNA Complex of the C-Terminal Domain of Ler

**DOI:** 10.1371/journal.ppat.1002380

**Published:** 2011-11-17

**Authors:** Tiago N. Cordeiro, Holger Schmidt, Cristina Madrid, Antonio Juárez, Pau Bernadó, Christian Griesinger, Jesús García, Miquel Pons

**Affiliations:** 1 Institute for Research in Biomedicine (IRB Barcelona), Parc Científic de Barcelona, Barcelona, Spain; 2 Max Planck Institute for Biophysical Chemistry, Department of NMR-based Structural Biology, Göttingen, Germany; 3 Department of Microbiology, University of Barcelona, Barcelona, Spain; 4 Institut de Bioenginyeria de Catalunya (IBEC), Parc Científic de Barcelona, Barcelona, Spain; 5 Department of Organic Chemistry, University of Barcelona, Barcelona, Spain; Tufts University School of Medicine, United States of America

## Abstract

Ler, a member of the H-NS protein family, is the master regulator of the LEE pathogenicity island in virulent *Escherichia coli* strains. Here, we determined the structure of a complex between the DNA-binding domain of Ler (CT-Ler) and a 15-mer DNA duplex. CT-Ler recognizes a preexisting structural pattern in the DNA minor groove formed by two consecutive regions which are narrower and wider, respectively, compared with standard B-DNA. The compressed region, associated with an AT-tract, is sensed by the side chain of Arg90, whose mutation abolishes the capacity of Ler to bind DNA. The expanded groove allows the approach of the loop in which Arg90 is located. This is the first report of an experimental structure of a DNA complex that includes a protein belonging to the H-NS family. The indirect readout mechanism not only explains the capacity of H-NS and other H-NS family members to modulate the expression of a large number of genes but also the origin of the specificity displayed by Ler. Our results point to a general mechanism by which horizontally acquired genes may be specifically recognized by members of the H-NS family.

## Author Summary

Pathogenic *Escherichia coli* strains and other enterobacteria carry genes acquired from other bacteria by a process known as horizontal gene transfer. Proper regulation of the genes that are expressed in a given moment is crucial for the success of the bacteria. The protein H-NS is a global regulator that binds DNA and maintains a large number of genes silent until they are required, for example, to sustain the bacteria's colonization of a new host. Ler is a member of the H-NS family that competes with H-NS to activate the expression of a group of horizontally acquired genes that encode for a molecular machine used by *E. coli* to infect human cells. Ler and H-NS share a similar DNA-binding domain and can bind to different DNA sequences. Here, we present the structure of a complex between the DNA-binding domain of Ler and a natural DNA fragment. This structure reveals that Ler recognizes specific DNA shapes, explaining its capacity to regulate genes with different sequences. A single arginine residue is key for the recognition of a DNA narrow minor groove, which is one of, though not the only, hallmarks of the DNA shapes that are recognized by H-NS and Ler.

## Introduction

Enteropathogenic *Escherichia coli* (EPEC) and enterohaemorrhagic *E. coli* (EHEC) are causal agents of infectious diarrhea. While the former is responsible mainly for infantile diarrhea, EHEC infections are associated with hemorrhagic colitis and may produce a life-threatening complication known as hemolytic uremic syndrome. EPEC and EHEC are non-invasive pathogens that produce characteristic attaching and effacing (A/E) intestinal lesions [1]. The genes required for the formation of A/E lesions are clustered on a pathogenicity island known as the locus of enterocyte effacement (LEE). LEE genes are organized in five major operons (*LEE1* to *LEE5*) and several smaller transcriptional units and they encode the components of a type III secretion system (TTSS), an adhesin (intimin) and its receptor (Tir), effector proteins secreted by the TTSS, chaperones, and several transcription regulators [2]. The first gene of the *LEE1* operon encodes the LEE-encoded regulator Ler, which is essential for the formation of A/E lesions in infected cells [3], [4] and for the *in vivo* virulence of A/E pathogenic *E. coli* strains [5].

Ler (123 amino acids, 14.3 kDa) is the master regulator of LEE expression and is required to activate LEE genes that are otherwise repressed by the histone-like nucleoid structuring protein H-NS [2].

The H-NS protein, best characterized in *E. coli* and *Salmonella*, is a member of a family of transcriptional regulators with affinity for AT-rich DNA sequences that mediate the adaptive response of bacterial cells to changes in multiple environmental factors associated with colonization of different ecological niches, including human hosts. H-NS is usually an environmentally-dependent transcriptional repressor. H-NS-mediated repression (usually termed silencing) is alleviated either by alterations in physicochemical parameters (i.e., a transition from low (25°C) to high (37°C) temperature), by the activity of proteins that displace H-NS from its target DNA sequences, such as Ler, or by a combination of both. H-NS regulation is strongly associated with pathogenicity, thus understanding the basis of the selective regulation of virulence genes could lead to sustainable antimicrobial strategies that are less susceptible to acquiring resistance.

In addition to the LEE genes, Ler is also involved in the regulation of other horizontally acquired virulence genes located outside the LEE loci and scattered throughout the chromosome of A/E pathogenic strains [3], [6], [7]. However, Ler does not regulate other H-NS-silenced operons such as *bgl* [8] and *proU* [3]. This observation shows that Ler is not a general antagonist of H-NS, but a specific activator of virulence operons acquired by horizontal transfer (HT). Selective regulation of HT genes has been demonstrated in the plasmid R27 encoded H-NS paralogue (H-NS_R27_) and in chromosomal H-NS in the presence of a co-regulator of the Hha/YmoA family [9].

The mechanism of Ler-mediated activation has been extensively studied in operons located both within the LEE loci, such as *LEE2*/*LEE3* [10], *grlRA* [11], [12] and *LEE5* [8], and outside, including *nleA* (for non-LEE-encoded effector A) [13] and the *lpf1* fimbrial operon [6], [14]. These studies suggest that Ler counteracts the silencing activity of H-NS by directly binding to DNA and displacing H-NS from specific promoter regions. Ler does not exert dominant negative effects on H-NS function and there is no evidence of a direct interaction between Ler and H-NS [8]. Despite the wealth of biochemical/biophysical data, including the proposal of a DNA sequence consensus motif for H-NS [15], the lack of structural data on the complexes formed between H-NS or H-NS family members and DNA has until now prevented a detailed understanding of the mechanism of DNA recognition and the basis of the selectivity within H-NS family proteins.

All H-NS-related proteins identified to date are predicted to be organized in two structurally different domains. While the oligomerization domains of Ler and H-NS differ greatly, their DNA binding domains are very similar, thereby suggesting that they account for the similar recognition properties of both proteins, and possibly also for their distinct selectivity. While a possible interplay between protein oligomerization and DNA binding cannot be ruled out, a detailed understanding of the recognition mechanism by individual DNA-binding domains is a prerequisite for further studies.

The C-terminal domain of Ler (CT-Ler), exhibits significant amino acid homology with the C-terminal H-NS DNA-binding domain (CT-H-NS; 36.0% identity, 63.8% similarity) and its deletion abolishes DNA binding [16]. CT-Ler contains a sequence (TWSGVGRQP) similar to the consensus core DNA-binding motif found in H-NS-like proteins (TWTGXGRXP) [17]. Here we present the solution structure of a complex formed by CT-Ler bound to a natural occurring DNA sequence of the *LEE2*/*LEE3* regulatory region. This is the first report of a DNA complex that includes a member of the H-NS family characterized at atomic detail. Our results reveal that CT-Ler does not participate in base-specific contacts but recognizes specific structural features in the DNA minor groove. The indirect readout mechanism can be extended to H-NS and other H-NS family members and explains their capacity to modulate the expression of a large number of genes. The CT-Ler/DNA structure provides clues for the mechanism by which HT genes may be specifically recognized by members of the H-NS family and illustrates the general features of DNA minor groove readout.

## Results

### CT-Ler/DNA complex formation

**Figure 1:**
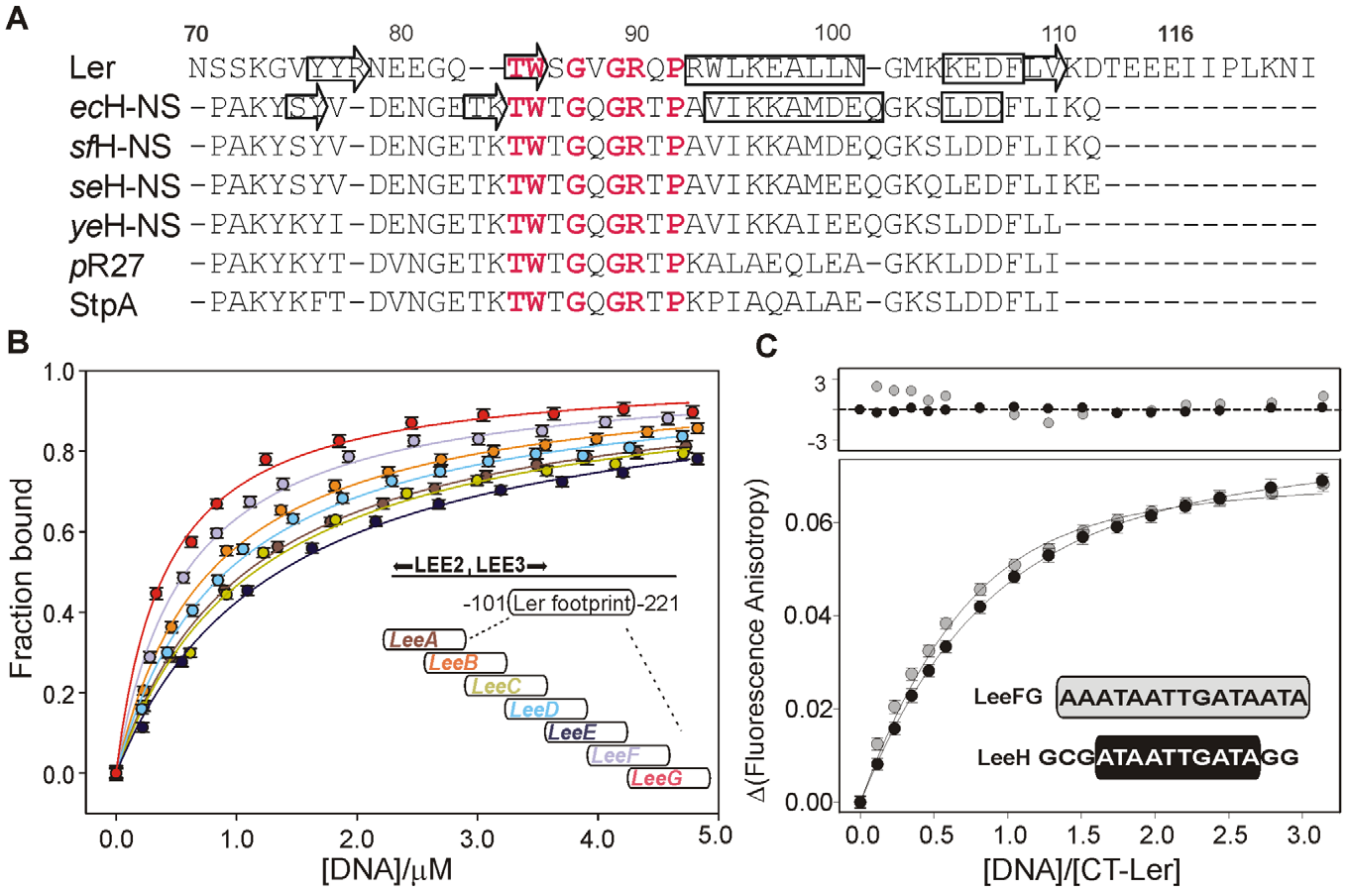
DNA-binding domain of selected members of the H-NS family of proteins and DNA fragment optimization. (**A**) Sequence alignment of the C-terminal domain of the following proteins: Ler; chromosomal H-NS of *E. coli* (ecHNS); *Shigella flexneri* (sfHNS); *Salmonella enterica* serovar Typhimurium (seHNS); *Yersinia enterocolitica* (yeHNS); the plasmid R27-encoded H-NS protein (pR27); and *E. coli* StpA. The secondary structure elements of DNA-bound CT-Ler and free H-NS are shown. Highly conserved residues within the consensus DNA-binding motif are highlighted in red. (**B**) Analysis of the interaction of CT-Ler with 30 bp DNA fragments (LeeA-G, sequences are listed in Table S1) derived from the DNAse I footprint of Ler in the *LEE2*/*LEE3* regulatory region [10]. Complex formation was followed by the increase of CT-Ler fluorescence anisotropy. (**C**) Fluorescence anisotropy titrations of CT-Ler with LeeH (black circle) and LeeFG (gray circle). Solid curves are the best fit to a model assuming a 1∶1 complex. The point by point deviations between fitting and experimental points are shown in the top panel.

We used a CT-Ler construct encompassing residues 70–116 (Figure 1A). This construct gave rise to a folded and functional domain (Figure S1) with excellent solubility and long-term stability. Residues 117–123 are part of an extension that is dispensable to counteract H-NS repression [18]. NMR spectra of a construct including these residues showed that they are disordered and have no effect on the structure of the folded domain, as seen by the exact coincidence of the cross-peak position of most residues in HSQC NMR spectra of different constructs (Figure S2).

The sequence of the short DNA fragment used to form the complex was based on the regulatory region of the *LEE2/LEE3* operons spanning positions -221 to -101. This region was protected by Ler in footprinting experiments [10]. Seven 30 bp long dsDNA, LeeA-LeeG, with a 15 bp overlap between consecutive fragments (Figure 1B, Table S1) were tested for binding to CT-Ler using fluorescence anisotropy. As positive and negative controls, we used two 30-mer duplexes: an adenine tract that was previously employed to study the DNA-binding properties of CT-H-NS, (GGCAAAAAAC)_3_ [19] and (GTG)_10_ (Figure S3). CT-Ler showed the highest affinities for LeeF and LeeG (Figure 1B) and we further analyzed its binding to the 15 bp overlapping region of theses two fragments, namely LeeFG (AAATAATTGATAATA). Fluorescence anisotropy titrations showed small but systematic deviations from the 1∶1 model, suggesting simultaneous multiple binding to this DNA sequence (Figure 1C). Since the consensus binding motif proposed for H-NS is only 10 bp long [15] we designed a new 15 bp DNA, LeeH (GCGATAATTGATAGG), containing the central 10 bp of LeeFG flanked by GC base pairs for thermal stability. LeeH partially matches the proposed H-NS consensus sequence (tCG(t/a)T(a/t)AATT) [15]. A good fit to a 1∶1 model with apparent *K_d_* 1.10±0.05 µM was observed for this duplex (Figure 1C).

### Structure of the CT-Ler/DNA complex

The complex of CT-Ler with LeeH was solved by a combination of NMR and small-angle X-ray scattering (SAXS). The structure determination protocol consisted of the independent calculation of the structure of bound CT-Ler and DNA, followed by intermolecular NOE (*i*NOEs) driven docking and a final scoring including SAXS data. CT-Ler structures were calculated based on 1302 NOE distance restraints, together with torsion angle and experimentally determined hydrogen bonds. The restraint and structural statistics of the 20 lowest energy structures are shown in Table S2. None of the structures contained distance or dihedral angle violations >0.5 Å or 5°, respectively.

The pattern and intensities of bound DNA NOEs were typical of a B-form. The DNA structure was optimized in explicit solvent using experimental restrains determined in the bound form, starting from canonical B-DNA as described in the Materials and Methods section. The absence of major distortions in the DNA structure caused by CT-Ler binding was confirmed by the good agreement between the experimental SAXS curve of free LeeH and the prediction based on the DNA model extracted from the final complex (Figure S4).

**Figure 2:**
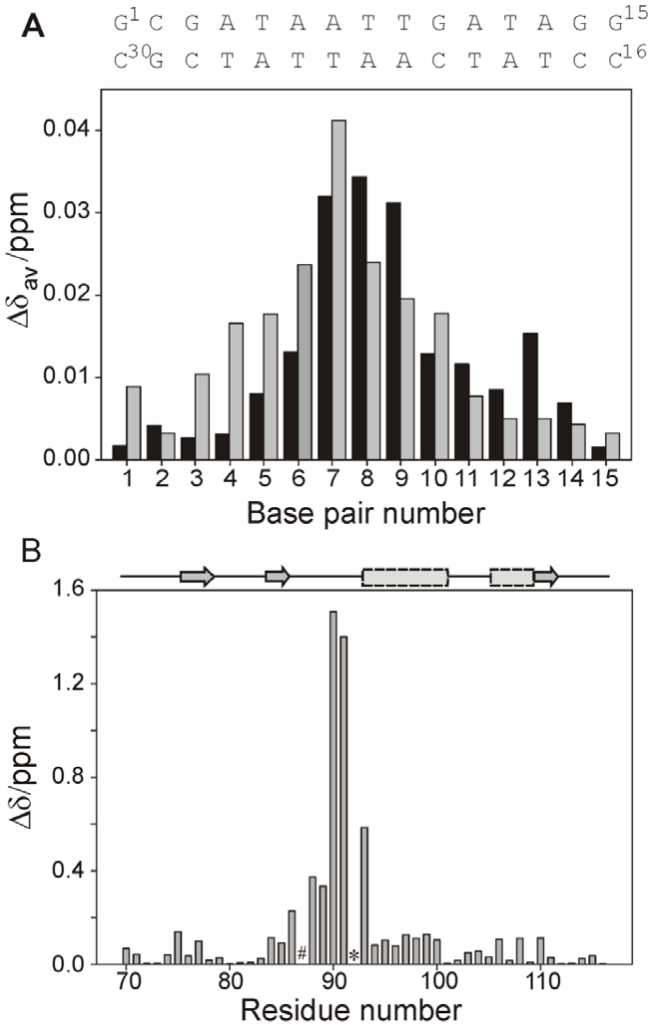
NMR analysis of the CT-Ler/LeeH interaction. (**A**) Mean absolute changes in ^1^H-NMR chemical shifts caused by the addition of 0.5 equivalents of CT-Ler. The average is over all resolved resonances per nucleotide. The upper and lower LeeH strands are identified by black and gray bars, respectively. (**B**) Backbone amide chemical shift changes in CT-Ler (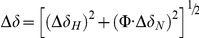) upon complex formation with LeeH. The scaling factor 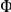 corresponds to the ratio of ^15^N and ^1^H magnetogyric constants. Resonances that were not observed are denoted by # (Gly87) or * (Pro92).

**Figure 3:**
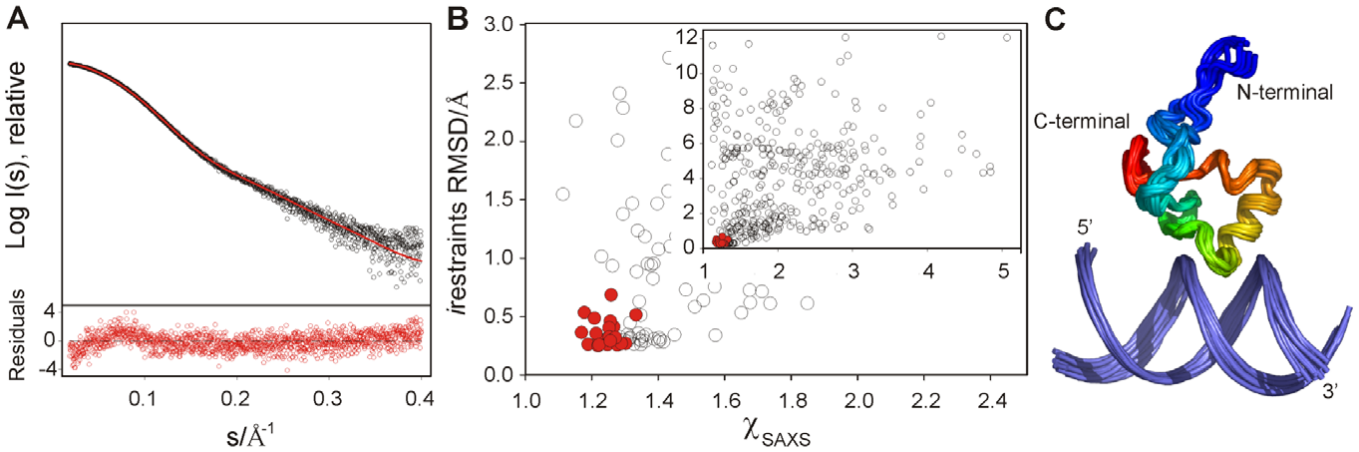
Structure determination of the CT-Ler/LeeH complex based on NMR and SAXS. (**A**) SAXS intensity in logarithmic scale measured for a CT-Ler/LeeH equimolar sample (open circles) as a function of the momentum transfer 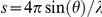, where 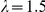 Å is the X-ray wavelength and 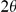 is the scattering angle. CRYSOL fit of the SAXS curve using a representative NMR structure (red); the average deviation 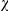 is 1.16. Only the range 0.018< *s* <0.4 Å^−1^ is displayed. The point by point deviations [(I(*s*)^exp^−I(*s*)^fit^)/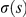], where 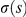 is the experimental error are shown in the bottom panel. (**B)** Scatter plot of NMR intermolecular restraint violations versus 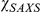 values for the initial set of 400 complex structures and the final ensemble of 20 low energy structures highlighted in red (inset). The main panel shows a zoom of the best structures. (**C**) Backbone overlap of the 20 lowest energy complex structures. Protein backbone is coloured in rainbow gradation.

The DNA region most affected by CT-Ler binding, identified by the combined chemical shift perturbations of nucleotide protons, is centered in the symmetrical 4 bp AT-tract, AATT (Figure 2A). The largest chemical shift perturbations of CT-Ler (Figure 2B) were observed for residues Val88 to Arg93. The 30 assigned *i*NOEs involve protein residues located in the region where the chemical shift perturbations were observed. On the basis of these *i*NOE restraints and the mapped interfaces, 400 CT-Ler/LeeH complex structures were generated as described in Materials and Methods and ranked by energy and NMR intermolecular restraint (*i*restraint) violations. The quality of the structures was confirmed by comparing the predicted and experimentally determined SAXS curves of the complex. The SAXS profile predicted for the best NMR-derived complex structure is in good agreement with the experimental curve (Figure 3A). The scatter plot in Figure 3B shows that, in general, the best NMR structures also fit SAXS data well. The final ensemble of 20 structures was selected using a scoring function that combined docking energy and measures of the agreement with experimental NMR and SAXS data (red circles). The ensemble is well defined (Figure 3C), with a pairwise RMSD (heavy atoms) of 1.30±0.38 Å and all conformers exhibited good geometry, no violations of *i*NOE distance restraints >0.5 Å and correctly explained the SAXS data. Most of the protein residues are in the core region of the Ramachandran plot. The small *i*restraint deviations illustrate that the protein-DNA interface is well defined, allowing us to elucidate a molecular basis for CT-Ler/LeeH recognition.

The structure of DNA-bound CT-Ler contains a central helix (residues 93–101) and a triple-stranded antiparallel β-sheet (β1:76–78, β2:84–85, β3:109–110). The β1-β2-hairpin is connected to the α-helix by a loop (Loop2:86–92). A turn and a short 3_10_-helix (105–108) link the helix to the β3 strand. The similarity between the C^α^ and C^β^ secondary chemical shifts of the free and bound forms indicate that the secondary structure is retained upon binding (Figure S5). The overall protein fold is analogous to that previously described for CT-H-NS in the absence of DNA [19].

**Figure 4:**
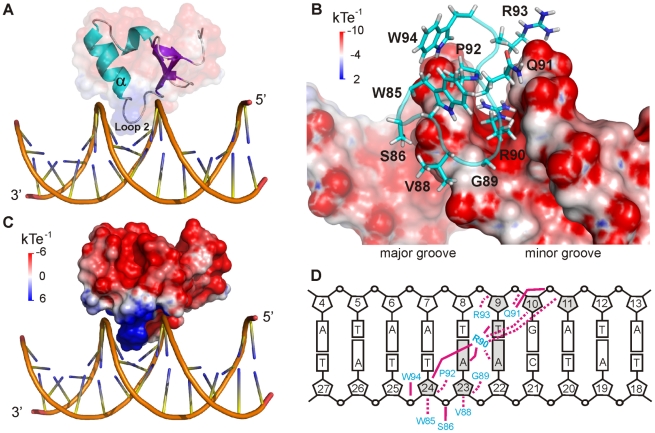
CT-Ler/LeeH interactions. (**A**) Structure of CT-Ler/LeeH complex. CT-Ler is shown as a ribbon diagram and transparent surface representation. Interactions involve the DNA minor groove and Loop2 and the α–helix of CT-Ler. (**B**) Close-up view of the binding interface. CT-Ler residues involved in DNA recognition are shown as stick models. The electrostatic potential of LeeH, calculated with DelPhi in the absence of CT-Ler, is shown. (**C**) Electrostatic potential of CT-Ler. The orientation of the complex is the same as in A. (**D**) Schematic representation of the hydrophobic (dashed lines) and polar (solid lines) intermolecular contacts.

CT-Ler binds as a monomer inserting Loop2 and the N-terminal end of the α-helix into the DNA minor groove and contacting the central 6 bp region (A^6^A^7^T^8^T^9^G^10^A^11^) (Figure 4). The complex buries 953±55.64 Å^2^ of surface area and is stabilized by non-specific hydrophobic and polar contacts, involving mainly the sugar-phosphates backbone and residues of the consensus DNA-binding motif found in H-NS-like proteins. Residues Trp85, Gly89, Arg90 and Pro92 (Figure 1A), highly conserved among H-NS-like proteins, are located in the complex interface (Figure 4B), and all gave rise to *i*NOE restraints with DNA. A summary of the observed intermolecular contacts is shown in Figure 4D.

The interaction surface of CT-Ler is positively charged and the Arg90 side chain is deeply inserted inside a narrow minor groove (Figure 4B and C). In addition, Arg93 at the N-terminus of the α-helix and the helix-dipole moment itself create a positively charged region that points into the negatively charged minor groove.

**Figure 5:**
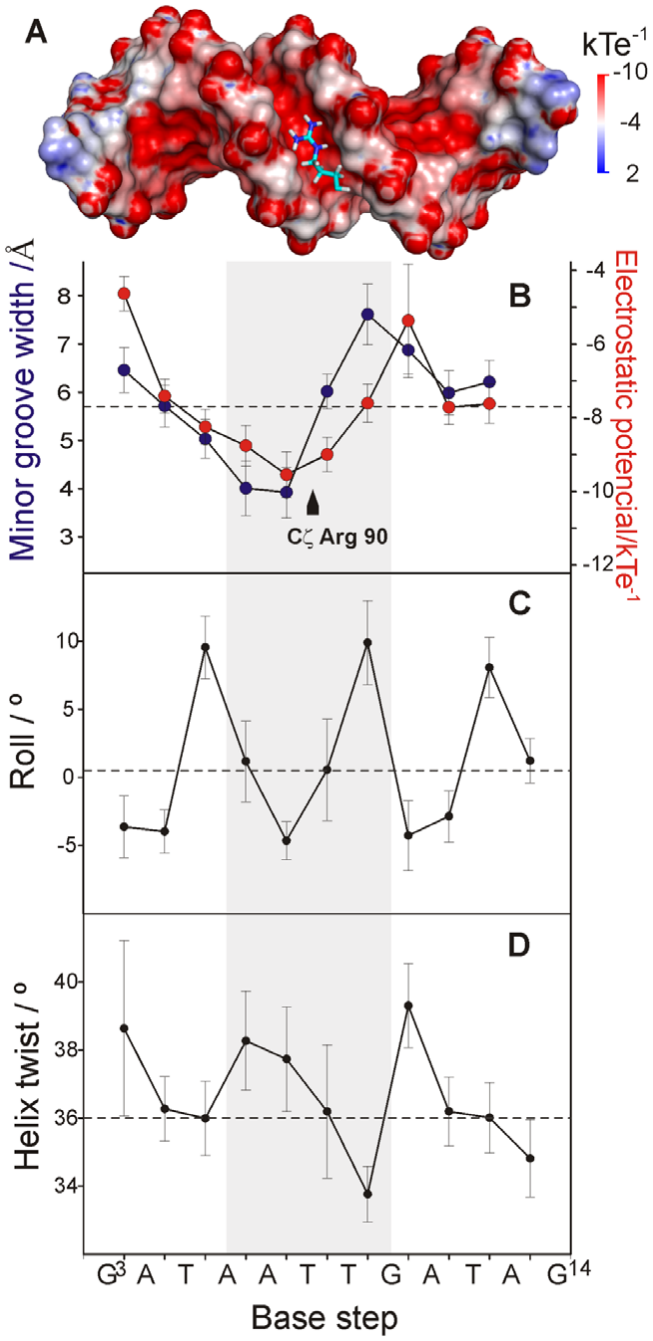
DNA recognition by CT-Ler is dictated by the minor groove width. (**A**) Stick representation of Arg90 side chain inserted at the floor of the negatively charged LeeH minor groove. The electrostatic potential of LeeH, calculated in the absence of CT-Ler, is plotted on the LeeH surface. (**B**) Average minor-groove width (blue) and electrostatic potential in the centre of LeeH minor groove (red). The position of the guanidium group of Arg90 is indicated. (**C-D**) Helical parameters of LeeH in complex with CT-Ler. Roll and helix twist angles are shown. Dashed lines correspond to values typical of canonical B-DNA [56].

The width of the LeeH minor groove varies along the sequence and deviates significantly from the average value of canonical B-DNA (Figure 5). The groove progressively narrows towards the A^7^pT^8^ base step, and widens at the T^9^pG^10^ base step. The DNA electrostatic potential is modulated by the width of the minor groove. The guanidinium group of Arg90 interacts with the narrowest region of the groove where the electrostatic potential is most negative (Figure 5A and B). The approach of Loop2, where Arg90 is located, is enabled by the adjacent widening of the minor groove.

Sequence-dependent variations of DNA structure can be described in terms of helical parameters, such as roll and helix twist (Figure 5C and D). The roll angle is most negative (−4.64°±1.38) at the A^7^pT^8^ base step and is small or negative for most of the steps in LeeH except for the pyrimidine-purine base steps, which show large positive values. A series of consecutive small/negative roll angles leads to the narrowing of the minor groove [20]. The groove widening at T^9^pG^10^ can be traced to a combination of positive roll and a small helix twist of 33.8°±0.8, indicating that the segment is slightly unwound with respect to the standard B-form. The region including the A^6^A^7^T^8^T^9^ stretch is slightly overwound, with an average helix twist of 37.4°±1.6.

### Arg90 is essential for Ler binding

**Figure 6:**
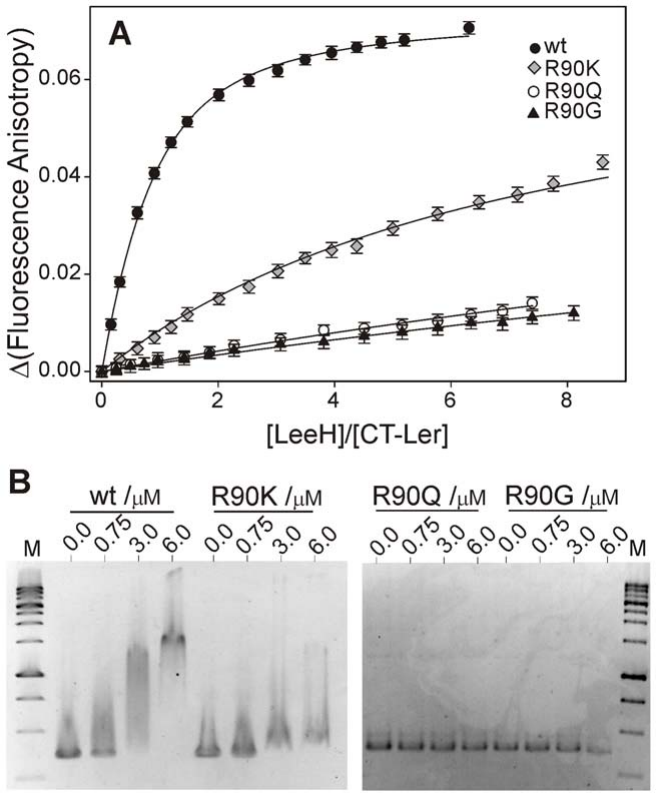
Arg90 is essential for DNA-binding. (**A**) Fluorescence anisotropy titrations of wild type, R90K, R90Q and R90G CT-Ler with LeeH. (**B**) EMSA of wild type and mutant Ler proteins. 80 ng of DNA (*LEE2* positions −225 to +121) were incubated with the indicated Ler concentrations and analyzed on a 1.5% agarose gel. 1 Kb DNA ladder was included as a reference (lane M).

To verify the relevance of Arg90 in the interaction, we replaced this residue by glycine (R90G), glutamine (R90Q) or lysine (R90K) and tested their effects on the affinity of CT-Ler to LeeH. All CT-Ler variants were properly folded, as determined from NMR, and their interaction with LeeH was measured by fluorescence anisotropy (Figure 6A). The mutated domains showed no affinity to LeeH or highly reduced affinity (R90K), thereby confirming that Arg90 is an essential residue.

The effect of these mutations on the binding of Ler(3–116), including the oligomerization domain, to the *LEE2* regulatory region (positions −225 to +121) was determined using electrophoretic mobility shift assays (EMSA) (Figure 6B). In agreement with the results obtained with the isolated CT-domain, DNA binding by Ler is abolished by R90Q and R90G mutations and strongly reduced in the case of the R90K variant. These experiments confirm the essential role of Arg90 in the context of the oligomeric Ler protein and for the range of binding sequences present in one of its natural targets.

### DNA sequence specificity of Ler binding

The structure of the CT-Ler/LeeH complex does not show base specific contacts. On the contrary, the structure of the complex suggests that CT-Ler recognizes local structural features of the minor groove that may be associated with distinct DNA sequences. In order to gain some insight into the range of DNA sequences that can be recognized by CT-Ler, we measured the dissociation constants of complexes formed by two series of short DNA duplexes related to the LeeH sequence. In the first series we introduced a single base pair replacement in each of the ten central positions of LeeH. Adenines and thymines were replaced by guanines and cytosines, respectively, and guanine in position 10 was mutated to adenine, to preserve the purine-pyrimidine sequence. In the second series, we compared the binding of CT-Ler to several 10-mer duplexes. One of these contained the AT-tract (AATT) that interacts with CT-Ler in the LeeH complex flanked by GC base pairs to ensure thermal stability. Variants were designed to test the effect of interrupting the AT-tract by TpA steps at a number of positions.

**Figure 7:**
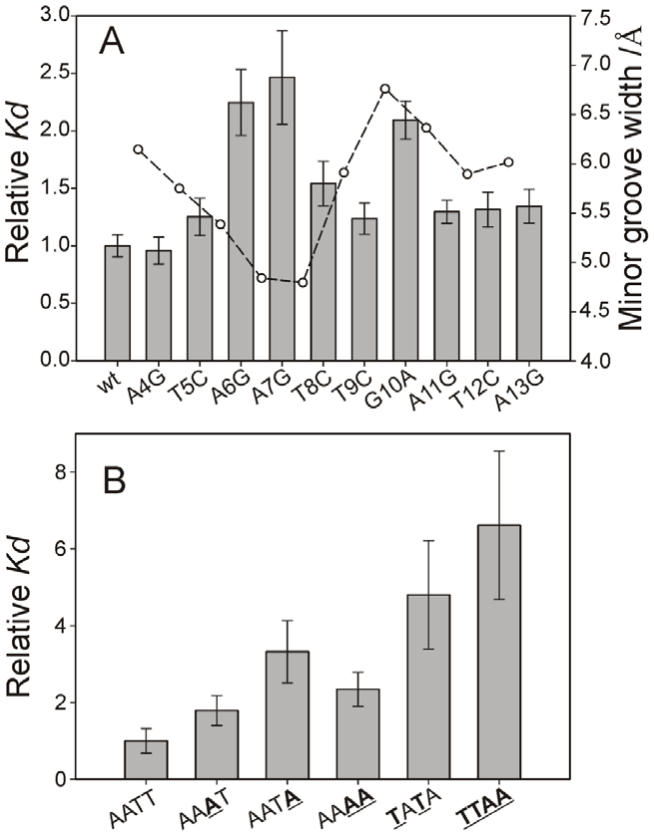
Minor groove shape serves as a signature for CT-Ler/DNA recognition. (**A**) CT-Ler binding to DNA variants containing single base-pair substitutions with respect to LeeH (wt). The LeeH minor groove width is also shown to highlight the fact that mutations in the compressed and expanded regions of the minor groove caused the largest effects. (**B**) Relative *Kd* values of the complexes formed between CT-Ler and 10-mer duplexes with different AT-rich sequences. The most stable complex, used as reference, has the AATT sequence present in LeeH. Relative *Kd* values are *Kd*(mutant)/*Kd*(reference) determined by fluorescence anisotropy.

Affinity to CT-Ler was measured by fluorescence anisotropy. The results are shown in Figure 7 and the DNA sequences and dissociation constants are listed in Table S3.

Figure 7A shows the relative *K_d_* values of the single base-pair replacements of LeeH. The largest effects were observed when the base pairs of A^6^ or A^7^ were replaced. The base pair of G^10^ resulted to be similarly relevant. A smaller effect was observed at the position of T^8^. Small non-specific effects were observed in all the remaining sites except that of A^4^. The most affected base pairs were at the sites where the minor groove width in LeeH is more different from the standard B-DNA and define the features that we hypothesize to be recognized by CT-Ler: the narrow groove where the Arg90 side chain is inserted and the wide adjacent region that enables the approach of Loop2.

Figure 7B show the relative dissociation constants of the complexes formed by the 10-mer duplexes. The presence of TpA steps in CGCAA**TA**GCG, CGC**TATA**GCG and CGCT**TA**AGCG results in a decrease in the stability of the complexes. The remaining three sequences (CGCAATTGCG, CGCAAATGCG, and CGCAAAAGCG) show AT-tracts of the same length but their affinity for CT-Ler differs. The complex with the A_4_ stretch is 2-fold less stable than that containing the AATT motif.

The AT-tract in LeeH is terminated by a TpG pyrimidine-purine step. Replacing it by a TpC pyrimidine-pyrimidine step in a 10 bp duplex had only a minor effect on the affinity for CT-Ler (*cf.* AATT and AATTC in Table S3). Interestingly, replacement of the T^9^pG^10^ step in LeeH by the alternative pyrimidine-purine step, TpA, resulted in a major loss of stability of the complex.

### CT-Ler provides insight into DNA binding by H-NS

The DNA binding domains of Ler and H-NS share a high degree of similarity both in sequence and in structure. We carried out experiments to specifically test two key points that are apparent from the analysis of the Ler/LeeH complex, namely the role of the conserved arginine residue (Arg90 in Ler, Arg114 in H-NS) in Loop2 and the requirement for an AT-tract and the effect of interrupting TpA steps.

**Figure 8:**
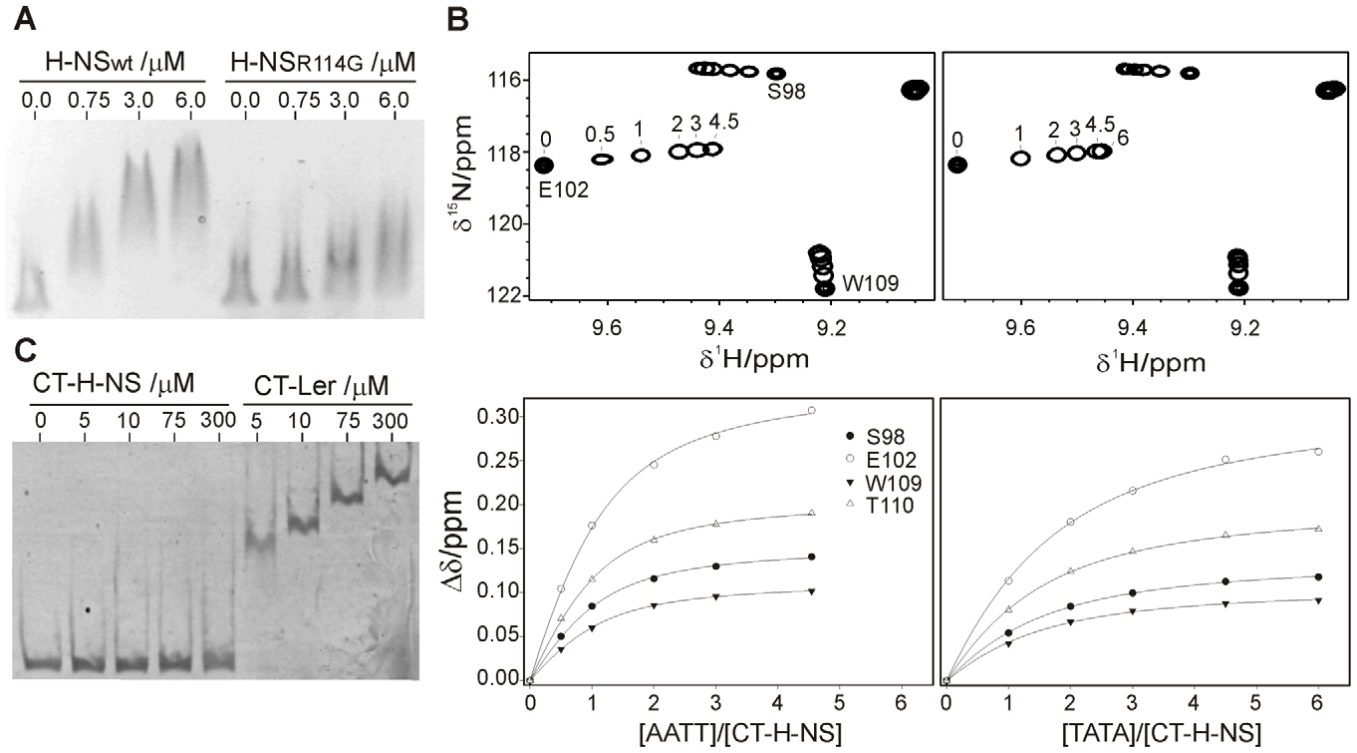
The DNA-binding domains of Ler and H-NS share a similar indirect DNA readout mechanism. (**A**) EMSA (1.5% agarose) of the −225 to +121 *LEE2* fragment (80 ng) with increasing concentrations of wild type and R114G H-NS proteins. (**B**) DNA titrations of CT-H-NS followed by NMR. Expansions of ^1^H-^15^N HSQC spectra of CT-H-NS in the presence of the 10 bp duplexes AATT (top left, 0, 0.5, 1, 2, 3 and 4.5 equivalents) or TATA (top right, 0, 1, 2, 3, 4.5 and 6 equivalents). The DNA-dependent shifts of selected cross-peaks were fitted to a 1∶1 model (bottom), supported by the strict linear displacement of the cross-peaks during the titration. (**C**) CT-Ler and CT-H-NS binding to the −225 to +121 *LEE2* fragment (20 ng) followed by EMSA on a 7% polyacrylamide gel.

H-NS Arg114, corresponding to Arg90 in Ler, was mutated to glycine and the affinity towards the −225 to +121 *LEE2* region was compared with that of the wild type form by EMSA. As in the case of Ler, replacing the arginine residue in Loop2 results in a substantial loss of affinity (Figure 8A). However, H-NS retains some residual activity even when arginine was replaced by glycine while this drastic mutation caused a complete loss of activity in the case of Ler.

The requirement for a narrow minor groove in the case of Ler can be assessed by the relative affinities towards the AATT and TATA 10-mer duplexes. Titrations of CT-H-NS with both oligonucleotides (Figure 8) provided dissociation constants of *circa* 41 μM for the AATT complex and 102 μM, 2–3-fold larger, for the TATA complex. CT-Ler showed similar relative affinities for the same oligonucleotides (Table S3), thereby suggesting that these two domains have similar requirements for a narrow minor groove.

As many H-NS and Ler target sequences may overlap, the relative affinity of the DNA-binding domains of these two proteins is relevant. As the CT-Ler complex studied included only the structured domain, we compared CT-Ler with the CT-domain of H-NS including only residues 95 to 137, excluding linker residues. This H-NS construct is properly folded as shown by the observation of well resolved NMR spectra (Figure 8). The same natural DNA fragment (*LEE2* positions −225 to +121) used in EMSA assays with Ler (Figure 6B) and H-NS (Figure 8A) was selected to compare the affinities of the CT-domains of these two proteins. The large number of binding sites for Ler and H-NS in this extended DNA fragment, as shown by footprinting experiments, allows the assessment of the relative overall affinities of the two domains for the whole range of sequences present in one of their common natural targets. The affinity of CT-Ler is larger than that of CT-H-NS, which under the conditions of the experiment hardly caused any retardation (Figure 8C). This observation contrasts with the similar affinity towards the same DNA fragment shown by longer constructs of Ler and H-NS that include the oligomerization and linker domains (cf. Figure 6B and 8C) and highlights varying relevance of interactions outside the folded CT-domains of these two proteins. The contribution of residues outside of the structured H-NS DNA-binding domain has been previously described [21], [22].

## Discussion

The structure of the complex between CT-Ler and LeeH shows that DNA shape and electrostatics, rather than base specific contacts, form the basis for the recognition of the CT-Ler binding site. This mechanism is referred to as indirect readout. Arg90 is a key residue for the CT-Ler interaction with DNA. Its side chain is inserted deep into a narrow minor groove. The requirement for Arg90 is strict in the case of CT-Ler and the R90G and R90Q mutants of Ler are totally inactive. The R90K mutant shows some residual binding suggesting that a positive charge is required. Arginine interactions with the DNA minor groove have been described in eukaryote nucleosomes [23], [24] and in DNA interactions by a nucleoid-associated protein of *Mycobacterium tuberculosis* [25]. These observations suggest that this mechanism may be universal for indirect DNA recognition of AT-rich sequences. A correlation between minor groove width and the electrostatic potential has been demonstrated as well as the preference for arginine binding to the narrowest regions where the electrostatic potential is more negative [23].

For CT-Ler, the narrow minor groove may be provided by a relatively short AT-tract as only the Arg90 side chain has to be inserted. The minimum width in the AATT motif is observed at the ApT step, matching the site where the guanidinium group is inserted. Continuous polyA tracts of 4 (Figure 7) and 6 nucleotides (Figure S3) of length give less stable complexes than sequences combining A and T. However, the presence of highly dynamic TpA steps [26] interrupting the AT-tracts decreases the affinity for CT-Ler. The presence of guanine, with its 2-amino group extending into the minor groove and increasing its width is also predicted to destabilize the insertion of the arginine side chain. We explored the effect of introducing TpG or TpA steps in the sequence recognized by CT-Ler. Figure 7 clearly shows that an uninterrupted AT-tract is needed for an efficient interaction with CT-Ler. However, a narrow AT-tract is not the only requirement for CT-Ler interaction. The lower affinity of the G10A variant of LeeH shows that, next to the narrow region, a rigid wide minor groove is also required to enable the access of Loop2 delivering the side chain of Arg90 into the narrowest region of the minor groove. Both sequences, T^9^pG^10^ in LeeH and T^9^pA^10^ in the mutated duplex, could adopt wide minor grooves. However, while the former is expected to provide a permanently wide groove, the flexible TpA step may switch between expanded and compressed forms, interfering with the approach of Loop2 directly or indirectly through the entropic penalty associated to stiffening of the DNA in the complex.

The structure of the complex as well as the affinity data with DNA sequence variants show that CT-Ler recognizes a pattern in the minor groove of DNA formed by two consecutive regions that are narrower and wider, respectively, with respect to standard B-DNA and show the optimal shape and electrostatic potential distribution for binding.

This structural pattern is present in the free LeeH DNA fragment as shown by the observation of diagnostic inter-strand NOES between AdeH2 and ThyH1' protons of A^7^/A^23^ and T^25^/T^9^, respectively supporting minor groove narrowing both in the free and bound forms of LeeH. Moreover, the SAXS data of free LeeH is better explained by the structure of LeeH in the complex than the structure of a canonical B-DNA LeeH (Figure S4). Therefore, at least in the case of LeeH, CT-Ler recognizes pre-existing DNA structural features following an indirect readout mechanism.

The molecular basis of the preference that H-NS displays for some promoter regions has been extensively studied. AT-tracts were initially postulated to be high affinity sites for H-NS and related to the presence of a narrow minor groove [27]. More recently, two short high affinity H-NS sites with an identical sequence, 5'-TCGATATATT-3' were identified in the *E. coli proU* promoter [28]. Lang et al. proposed that a 10 bp long consensus sequence (tCG(t/a)T(a/t)AATT) [15] acts as a nucleation site for cooperative binding to more extensive regions. In a recent study, a shorter segment of 5–6 nucleotides comprising only A/T nucleotides was found to be over-represented in genomic loci bound by H-NS in *E. coli* [29]. The interaction of the H-NS CT-domain, including a few residues from the linker region, with a short oligonucleotide was studied by NMR [22]. The authors concluded that a structural anomaly in the DNA associated with a TpA step was crucial for H-NS recognition.

Our results suggest that AT-tracts and wide TpA steps may be simultaneously required by H-NS family proteins. The correct positioning of a compressed and widened minor groove is the specific recognition signal for CT-Ler. Pyrimidine-purine steps tend to widen the minor groove and TpA steps may contribute to its widening, which is required after the AT-tract. However, in the case of Ler, a TpG step was preferred to the TpA step, suggesting that a wide narrow groove after the AT-tract is the true structural requirement.

CT-Ler and CT-H-NS showed similar structural requirements: mutation of Arg114 reduced the affinity of the complex, and introduction of TpA steps in the AT-tract caused a similar decrease in stability. This result is consistent with the fact that Ler targets can also be occupied by H-NS. Ler and H-NS bind to multiple sites. An indirect readout mechanism allows recognition of multiple sequences, if they adopt similar minor groove patterns.

The absence of structural changes between the free and bound forms of CT-Ler (Figure S5) supports a lock and key model for interactions involving the structured CT-domain and may account for the relatively high specificity of Ler, as compared with H-NS where additional interactions outside the CT-domain are comparatively more important. Comparison of constructs containing exclusively the structured region of the CT-domains of Ler and H-NS show that the former has higher affinity for the range of sequences present in a natural segment where both proteins bind. Several features, not present in CT-H-NS, may contribute to the higher stability of the CT-Ler complex. An additional arginine residue (Arg93) combined with the helix dipole provides additional electrostatic interactions, thus stabilizing the CT-Ler complex. While both Ler and H-NS have a conserved tryptophan residue that, in the case of Ler, forms hydrophobic interactions with DNA, CT-Ler presents an additional tryptophan residue in close contact with DNA. The dipoles of both indole rings are oriented with their positive end towards the negatively charged DNA backbone and the side chain NH of Trp94 forms a hydrogen bond with the DNA backbone.

We have determined for the first time the structure of a complex formed by the DNA-binding domain of a member of the H-NS family. Our results highlight the similarities in the DNA recognition mechanisms used by CT-Ler and CT-H-NS but also evidence some differences that may contribute to the differential recognition of some genes by Ler and H-NS.

## Materials and Methods

### Samples preparation

DNA fragments containing the coding sequence of Ler residues 65–123, 70–116 (CT-Ler) and 3–116 fused to an N-terminal His_6_-tag were amplified by PCR from EHEC strain 0157:H7 and subcloned into the pHAT2 vector. To overexpress CT-H-NS, DNA encoding this fragment (amino acids 95–137) with six histidine residues tagged at its N terminus was amplified by PCR using the full length H-NS construction [30] as template and then subcloned into the pHAT2 vector. Point mutations were generated using the QuikChange site-directed mutagenesis kit (Stratagene).

Ler fragments 65–123, 70–116 and 3–116 and CT-H-NS were overexpressed in BL21(DE3) cells with overnight incubation at 15°C by induction with 0.5 mM IPTG when an O.D._600_ of 0.7 was reached. For ^15^N and/or ^13^C isotopic labeling, cells were grown in M9 minimal media containing ^15^NH_4_Cl and/or ^13^C-glucose. For 10% ^13^C enrichment we used a carbon source consisting of a 1∶10 mixture of ^12^C-glucose/^13^C-glucose [31], [32]. Cells were harvested by centrifugation, frozen and resuspended in 20 mM HEPES (pH 8.0), 1 M NaCl, 5 mM imidazol, 5% (v/v) glycerol, treated for 30 min with lysozyme and DNAse and sonicated (6×10 s on ice). After centrifugation, the His-tagged fusion proteins were isolated with Ni-NTA beads (Qiagen) and further purified by size exclusion chromatography on a Superdex 75 column in 20 mM sodium phosphate, 150 mM NaCl, 0.2 mM EDTA, 0.01% (w/v) NaN_3_ pH 5.7 or 20 mM sodium phosphate, 300 mM NaCl, 0.01% (w/v) NaN_3_ pH 7.5. The expression and purification procedure for full length H-NS has been previously described [30].

DNA samples were prepared by hybridization of complementary oligonucleotides purchased from Sigma-Aldrich. Quality control was assessed by MALDI-TOF mass spectrometry. Oligonucleotides were mixed in equimolar amounts and annealed by heating to 92°C for 4 min and slowly cooled to room temperature.

### Fluorescence anisotropy measurements

Changes in CT-Ler intrinsic fluorescence anisotropy were monitored upon DNA addition. All measurements were recorded on a PTI QuantaMaster spectrophotometer equipped with a peltier cell, using an excitation wavelength of 295 nm to selectively excite CT-Ler tryptophans and emission detection at 344 nm. Fluorescence measurements were performed in 40 mM HEPES (pH 7.5), 60 mM potassium glutamate, 0.01% (w/v) NaN_3_ at 20°C. More details on data acquisition and equipment settings were previously described [33]. For the initial screening of the -221 to -101 regulatory region of *LEE2*, the apparent fraction saturation of CT-Ler was used to infer about DNA binding preferences. To measure the affinity of CT-Ler for 15 bp and 10 bp DNA fragments, titrations were performed at least in duplicate. The fitting was performed assuming a 1∶1 binding using the following equations [34]:
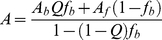
(1)

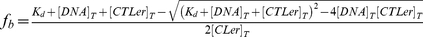
(2)where *A* is the observed anisotropy, *Af* and *Ab* are the anisotropies of free CT-Ler and the complex respectively, *f_b_* is the fraction of bound CT-Ler and *Q* is the ratio of quantum yields of bound and free forms. Equations 1 and 2 were solved iteratively until the theoretical binding isotherm matched the experimental data. *K_d_* and *A_b_* were considered to be adjustable parameters.

### NMR spectroscopy

All spectra were acquired at 25°C on 600, 700, 800 or 900 MHz Bruker spectrometers. Data processing and analysis were carried out with NMRPipe [35], NMRViewJ [36], and CARA [37].

NMR spectra for structure determination were recorded on a ∼1 mM sample containing a 1∶1 complex of uniformly ^13^C- and ^15^N-labeled CT-Ler and unlabeled DNA in 20 mM sodium phosphate (pH 5.7), 150 mM NaCl, 0.2 mM EDTA and 0.01% (w/v) NaN_3_. Backbone and aliphatic assignments of free and DNA-bound CT-Ler were obtained by standard methods. Aromatic resonances were assigned using 2D ^1^H-^13^C-edited-NOESY optimized for aromatic resonances. Stereospecific assignments of Val and Leu methyl groups were obtained from a constant time ^1^H-^13^C-HSQC on a 10% ^13^C-labeled protein sample [31]. Non-exchangeable protons of the LeeH duplex bound to CT-Ler were assigned using 2D F1,F2-^13^C-filtered TOCSY and NOESY spectra in D_2_O [38]. Exchangeable protons and H2 protons were assigned from 2D F1,F2-^15^N/^13^C-filtered NOESY spectrum in H_2_O [39]. Free DNA resonances were assigned using 2D DQF-COSY, TOCSY and 2D NOESY spectra. Proton chemical shifts were referenced using 4,4-dimethyl-4-silapentane-1-sulfonic acid (DSS) as an internal standard, whereas ^15^N and ^13^C chemical shifts were indirectly referenced. Chemical shift assignments have been deposited in the BioMagResBank database under BMRB accession number 17729.

Protein distance restraints were obtained from 2D ^1^H-^13^C-edited NOESY (aromatic optimized in D_2_O), 3D ^1^H-^15^N-edited NOESY-HSQC and two 3D ^1^H-^13^C-edited NOESY-HSQC (in H_2_O and in D_2_O) experiments with a mixing time of 120 ms. Data were automatically assigned and the NOE distance restraints were obtained iteratively using the Unio'08/CYANA 2.1 suite program [40], [41] and manually inspected. The distance restraints for the DNA in complex with CT-Ler were obtained measuring initial NOE build-up rates from 2D F1,F2-^15^N/^13^C-filtered NOESY spectra recorded with mixing time of 50, 75, 100 and 150 ms. Intermolecular NOEs were detected using a combination of 2D NOESY, 2D F1,F2-^13^C-filtered NOESY and 2D F2-^13^C-filtered NOESY experiments, together with 3D F1-^13^C,^15^N-filtered, [F2] ^13^C-edited 3D NOESY spectrum [42]. Additional intermolecular NOEs were obtained by analyzing the 3D ^15^N-edited and ^13^C-edited NOESY spectra.

Protein backbone dihedral angle restraints were derived using a combination of TALOS [43] and quantitative analysis of *^3^J_HNH_*_α_ obtained from a 3D HNHA spectrum [44]. Restraints on side chain 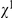 angle and stereospecific assignments of Hβ proton resonances were based on *^3^J_NH_*_β_ couplings, obtained from a 3D HNHB spectrum, in combination with observed intraresidual NOEs using the HABAS routine of the CYANA 2.1 program [45].

^1^H-^15^N HSQC spectra for analysis of the interaction of ^15^N-labeled CT-H-NS (100 µM) with dsDNA were obtained at 25°C in 20 mM sodium phosphate (pH 5.7), 150 mM NaCl, 0.2 mM EDTA and 0.01% (w/v) NaN_3_.

### Structure calculation and refinement

The structure of CT-Ler was determined by simulated annealing using the torsion angle dynamic simulation program CYANA 2.1 [45] and further water refinement with CNS 1.2.1 [46], [47]. Protein structure calculation was based on Unio'08/CYANA-generated upper distances, *^3^J_HNH_*_α_/*^3^J_NHH_*_β_ couplings, and TALOS-driven dihedral angle restraints. Based on H/D exchange experiments, backbone NOE pattern and ^13^C_α_/^13^C_β_ chemical shifts, hydrogen bond restraints were also used in the structure calculation. An ensemble of 100 protein structures was generated and the 20 lowest energy conformers were docked onto a B-DNA.

The observed overlap and broadening of DNA resonances hampered the complete quantitative analysis of NOESY spectra for bound DNA. Only a set of 282 well resolved cross-peaks were converted into distances using initial build-up rates and reference to the cytosine H5-H6 cross-peaks. Upper and lower limits were defined as ± 20% of the calculated distances. The structure of LeeH was fixed as B-DNA and further energy-refined using miniCarlo [48] followed by a 20 ps molecular dynamics refinement in explicit solvent using the Amber force field [49] and including NOE-derived distance restraints. To preserve the helical conformation of DNA, weak planarity restraints were also introduced. The DNA backbone was constrained to a range typical of B-form and all glycosidic angles were restrained as *anti*. Hydrogen bond restraints were used for all base pairs in which the imino proton was observed. The complex structure was generated employing 30 iNOEs, supplemented with highly ambiguous intermolecular restraints (AIRs) that were driven from the mapped binding interfaces. A total of 22 intermolecular NOE restraints were simultaneously assigned to the two symmetry-related protons in the AATT central region of the DNA and used as ambiguous restraints. HADDOCK 2.0 [50] was used to generated 2000 structures by rigid docking energy minimization, and 400 structures with the lowest energy were selected for semi-flexible refinement process. These 400 structures were finally refined in explicit water including all experimental restraints. Structures were then ranked using the energy-based HADDOCK scoring function (sum of intermolecular electrostatic, van der Waals, desolvation and AIR energies) and NOE energy term. The quality of these structures was evaluated in terms of the violations to the NOE data and the value 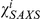 defining the agreement to SAXS curve. A final ensemble of 20 structures was obtained by re-scoring the pool of 400 structures using the following scoring function.
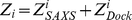
(3)

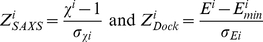
(4)where 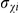 and 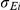 correspond to the root mean squared deviations with respect to the best possible value in 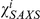 and *E^i^* respectively. Coordinates of the final ensemble were deposited in the Brookhaven Protein Data Bank under the accession number 2lev.

Minor groove geometry and helical parameters were analyzed using w3DNA [51]. Electrostatic potentials were obtained at physiological ionic strength using DelPhi [52].

### SAXS data collection and analysis

SAXS data for LeeH and the CT-Ler/LeeH complex were collected on a MAR345 image plate detector at the X33 European Molecular Biology Laboratory (DESY, Hamburg, Germany) [53]. The scattering patterns were measured at 25°C for 2 min at sample concentrations of 4.6 and 2.7 mg/ml and 6.6 and 3.3 mg/ml for LeeH and CT-Ler/LeeH, respectively. A momentum transfer range of 0.018< *s* <0.62 Å^−1^ was measured. Repetitive measurements indicated that samples did not present radiation damage. Buffer subtraction and the estimation of the radius of gyration, R_g_, and the forward scattering, I(0), through Guinier's approach were performed with PRIMUS [54]. The scattering profile of LeeH was obtained from merging curves at both concentrations. For CT-Ler/LeeH, SAXS profiles at both concentrations were virtually equivalent and only data from the highest concentrated sample were used for further analysis. Using Guinier's approach, the radii of gyration of LeeH and CT-Ler/LeeH were estimated to be 15.6±0.1 and 18.2±0.1 Å, respectively. All data manipulations were performed with the program PRIMUS. Using a bovine serum albumin sample (3.3 mg/ml), an estimated molecular weight of 18 kDa was obtained for CT-Ler/LeeH (theoretical MW of 16.3 kDa), thereby indicating the presence of a monomeric particle in solution. The agreement of the SAXS curve to various three-dimensional models was quantified with the program CRYSOL [55] using a momentum transfer range of 0.018< *s* <0.40 Å^−1^.

### Electrophoretic mobility shift assays

The DNA fragment used in this assay (*LEE2* positions −225 to +121) was obtained by PCR amplification from EHEC strain 0157:H7. The indicated concentrations of PCR-generated DNA and H-NS or Ler proteins were mixed in a total volume of 20 μl of 15 mM sodium phosphate, 100 mM NaCl, 0.01% (w/v) NaN_3_ pH 7.5. 1 mM tris(2-carboxyethyl)-phosphine (TCEP) was included for samples containing full length H-NS. After 20 min of incubation at room temperature, glycerol was added to 10% (w/v) final concentration and the reaction mixtures were electrophoresed on either 1.5% agarose or 7% polyacrylamide gels in 0.5x Tris-borate-EDTA buffer. The DNA bands were stained with ethidium bromide.

## Supporting Information

Figure S1 **Interaction between CT-Ler and the LeeH dsDNA fragment.** ^1^H-^15^N-HSQC NMR spectra of Ler70–116 (CT-Ler) recorded in the absence (black contours) and upon equimolar addition of LeeH (red contours). Side-chain NH groups are indicated by ‘sc’ after the residue number. The excellent chemical shift dispersion observed in the ^1^H-^15^N-HSQC NMR spectra indicates that the domain is properly folded.
(TIF)

Figure S2 **^1^H-^15^N-HSQC spectra of Ler fragments.** Overlap of ^1^H-^15^N-HSQC NMR spectra of Ler65–123 (green contours) and Ler70–116 (CT-Ler) (black contours) at pH 7.0 and 25°C. Most of the cross-peaks from CT-Ler coincide exactly with a cross-peak from Ler65–123. Additional residues from Ler65–123 show chemical shifts typical of unstructured residues.
(TIF)

Figure S3 **CT-Ler binds preferentially to AT-rich DNA sequences.** Fluorescence anisotropy titrations of CT-Ler with the following 30-mer duplexes: LeeG, (GGCAAAAAAC)_3_ and (GTG)_10_.
(TIF)

Figure S4 **Small-angle X-ray Scattering analysis of the 15** **bp LeeH duplex.** SAXS intensity in logarithmic scale measured for LeeH sample (open circles) as a function of the momentum transfer *s* = 4πsin(θ)/λ, where λ = 1.5 Å is the X-ray wavelength and 2θ is the scattering angle. Best CRYSOL fits to the curve using the structure of LeeH in the complex with CT-Ler (red) or the structure of a canonical B-DNA LeeH generated with w3dna (cyan); and the average deviations, χ, are 0.85, 1.27 respectively. Only the range 0.016< *s* <0.5 Å^−1^ is displayed. The point by point deviations for each fitting [(I(*s*)^exp^−I(*s*)^fit^)/σ(*s*)], where σ(*s*) is the experimental error are shown in the bottom panel with the same colour code.
(TIF)

Figure S5 **The CT-Ler secondary structure is not affected by DNA binding.** **(A)** Ribbon structure of a representative conformer of LeeH-bound CT-Ler. Elements of secondary structure are labeled on the structure. **(B)** Differences between the ^13^C^α^ (top panel) and ^13^C^β^ (bottom panel) chemical shifts observed for residues 70–116 of Ler and those expected for a random coil are plotted against the residue number. White and black bars correspond to the free and LeeH-bound forms, respectively.
(TIF)

Table S1 **DNA fragments used in the initial optimization of the CT-Ler/DNA complex.** DNA fragments span the Ler-footprint within the *LEE2*/*LEE3* regulatory region. Only the sequence of one of the complementary strands is shown.
(DOC)

Table S2 **NMR and refinement statistics.** Refinement statistics including the number and type of experimental restraints and the results of quality controls performed using PROCHECK [57] and CRYSOL [55].
(DOC)

Table S3 **DNA sequence effect on CT-Ler complex stability.** Sequences of the LeeH variants designed to test CT-Ler binding specificity and the corresponding dissociation constants. Only the sequence of one of the complementary strands is shown.
(DOC)

## References

[1] Croxen MA, Finlay BB (2010). Molecular mechanisms of *Escherichia coli* pathogenicity.. Nat Rev Microbiol.

[2] Mellies JL, Barron AM, Carmona AM (2007). Enteropathogenic and enterohemorrhagic *Escherichia coli* virulence gene regulation.. Infect Immun.

[3] Elliott SJ, Sperandio V, Girón JA, Shin S, Mellies JL (2000). The locus of enterocyte effacement (LEE)-encoded regulator controls expression of both LEE- and non-LEE-encoded virulence factors in enteropathogenic and enterohemorrhagic *Escherichia coli*.. Infect Immun.

[4] Friedberg D, Umanski T, Fang Y, Rosenshine I (1999). Hierarchy in the expression of the locus of enterocyte effacement genes of enteropathogenic *Escherichia coli*.. Mol Microbiol.

[5] Zhu C, Feng S, Thate TE, Kaper JB, Boedeker EC (2006). Towards a vaccine for attaching/effacing *Escherichia coli*: a LEE encoded regulator (*ler*) mutant of rabbit enteropathogenic *Escherichia coli* is attenuated, immunogenic, and protects rabbits from lethal challenge with the wild-type virulent strain.. Vaccine.

[6] Torres AG, López-Sánchez GN, Milflores-Flores L, Patel SD, Rojas-López M (2007). Ler and H-NS, regulators controlling expression of the long polar fimbriae of *Escherichia coli* O157:H7.. J Bacteriol.

[7] Abe H, Miyahara A, Oshima T, Tashiro K, Ogura Y (2008). Global regulation by horizontally transferred regulators establishes the pathogenicity of *Escherichia coli*.. DNA Res.

[8] Haack KR, Robinson CL, Miller KJ, Fowlkes JW, Mellies JL (2003). Interaction of Ler at the LEE5 (tir) operon of enteropathogenic *Escherichia coli*.. Infect Immun.

[9] Baños RC, Vivero A, Aznar S, García J, Pons M (2009). Differential regulation of horizontally acquired and core genome genes by the bacterial modulator H-NS.. PLoS Genet.

[10] Sperandio V, Mellies JL, Delahay RM, Frankel G, Crawford JA (2000). Activation of enteropathogenic Escherichia coli (EPEC) *LEE2* and *LEE3* operons by Ler.. Mol Microbiol.

[11] Barba J, Bustamante VH, Flores-Valdez MA, Deng W, Finlay BB (2005). A positive loop controls expression of the locus of enterocyte effacement-encoded regulatos Ler and GrlA.. J Bacteriol.

[12] Tauschek M, Yang J, Hocking D, Azzopardi K, Tan A (2010). Transcriptional analysis of the *grlRA* virulence operon from *Citrobacter rodentium*.. J Bacteriol.

[13] Schwidder M, Hensel M, Schmidt H (2011). Regulation of *nleA* in shiga toxin-producing *Escherichia coli* O84:H4 strain 4795/97.. J Bacteriol.

[14] Rojas-López M, Arenas-Hernández MMP, Medrano-López A, Martínez de la Peña CF, Puente JL (2011). Regulatory control of the *Escherichia coli* O157:H7 *lpf1* operon by H-NS and Ler.. J Bacteriol.

[15] Lang B, Blot N, Bouffartigues E, Buckle M, Geertz M (2007). High-affinity DNA binding sites for H-NS provide a molecular basis for selective silencing within proteobacterial genomes.. Nucleic Acids Res.

[16] Yerushalmi G, Nadler C, Berdichevski T, Rosenshine I (2008). Mutational analysis of the locus of enterocyte effacement-encoded regulator (Ler) of enteropathogenic *Escherichia coli*.. J Bacteriol.

[17] Bertin P, Hommais F, Krin E, Soutourina O, Tendeng C (2001). H-NS and H-NS-like proteins in Gram-negative bacteria and their multiple role in the regulation of bacterial metabolism.. Biochimie.

[18] Mellies JL, Larabee FJ, Zarr MA, Horback KJ, Lorenzen E (2008). Ler interdomain linker is essential for anti-silencing activity in enteropathogenic *Escherichia coli*.. Microbiology.

[19] Shindo H, Iwaki T, Ieda R, Kurumizaka H, Ueguchi C (1995). Solution structure of the DNA binding domain of a nucleoid-associated protein, H-NS, from *Escherichia coli*.. FEBS Lett.

[20] Haran TE, Mohanty U (2009). The unique structure of A-tracts and intrinsic DNA bending.. Q Rev Biophys.

[21] Shindo H, Ohnuki A, Ginba H, Katoh E, Ueguchi C (1999). Identification of the DNA binding surface of H-NS protein from *Escherichia coli* by heteronuclear NMR spectroscopy.. FEBS Lett.

[22] Sette M, Spurio R, Trotta E, Brandizi C, Brandi A (2009). Sequence-specific recognition of DNA by the C-terminal domain of nucleoid-associated protein H-NS.. J Biol Chem.

[23] Rohs R, West SM, Sosinsky A, Liu P, Mann RS (2009). The role of DNA shape in protein-DNA recognition.. Nature.

[24] West SM, Rohs R, Man RS, Honig B (2010). Electrostatic interactions between arginines and the minor groove in the nucleosome.. J Biomol Struct Dyn.

[25] Gordon BRG, Li Y, Wang L, Sintsova A, van Bakel H (2010). Lsr2 is a nucleoid-associated protein that targets AT-rich sequences and virulence genes in *Mycobacterium tuberculosis*.. Proc Natl Acad Sci USA.

[26] Travers AA (2004). The structural basis of DNA flexibility.. Phil Transat A Math Phys Eng Sci.

[27] Rimsky S, Zuber F, Buckle M, Buc H (2001). A molecular mechanism for the repression of transcription by the H-NS protein.. Mol Microbiol.

[28] Bouffartigues E, Buckle M, Badaut C, Travers A, Rimsky S (2007). H-NS cooperative binding to high-affinity sites in a regulatory element results in transcriptional silencing.. Nat Struct Mol Biol.

[29] Kahramanoglou C, Seshasayee AS, Prieto AI, Ibberson D, Schmidt S (2011). Direct and indirect effects of H-NS and FIS on global gene expression control in *Escherichia coli*.. Nucleic Acids Res.

[30] Nieto JM, Madrid C, Miquelay E, Parra JL, Rodríguez S (2002). Evidence for direct protein-protein interaction between members of the enterobacterial Hha/YmoA and H-NS family of proteins.. J Bacteriol.

[31] Neri D, Szyperski T, Otting O, Senn H, Wuthrich K (1989). Stereo-specific nuclear magnetic resonance assignments of the methyl groups of valine and leucine in the DNA-binding domain of the 434 Repressor by biosynthetically directed fractional ^13^C labeling.. Biochemistry.

[32] Szyperski T, Neri D, Leiting B, Otting G, Wüthrich K (1992). Support of 1H NMR assignments in proteins by biosynthetically directed fractional ^13^C-labeling.. J Biomol NMR.

[33] Cordeiro TN, García J, Pons JI, Aznar S, Juárez A (2008). A single residue loop mutation enhancing Hha binding to nucleoid associated protein H-NS results in loss of Hha regulatory propertie*s*.. FEBS Lett.

[34] Roehrl M, Wang J, Wagner G (2004). A general Framework and data analysis of competitive high-throughput screens for small-molecule inhibitors of protein-protein interactions by fluorescence Polarization.. Biochemistry.

[35] Delaglio F, Grzesiek S, Vuister GW, Zhu G, Pfeifer J (1995). NMRPipe: A multidimensional spectral processing system based on UNIX pipes.. J Biomol NMR.

[36] Johnson BA (2004). Using NMRView to visualize and analyze the NMR spectra of macromolecules.. Methods Mol Biol.

[37] Keller RLJ (2004). The Computer Aided Resonance Assignment Tutorial..

[38] Iwahara J, Wojciak JM, Clubb RT (2001). Improved NMR spectra of a protein-DNA complex through rational mutagenesis and the application of a sensitivity optimized istope-filtered NOESY experiment.. J Biomol NMR.

[39] Ikura M, Bax A (1992). Isotope-filtered 2D NMR of a protein-peptide complex: study of a skeletal muscle myosin light chain kinase fragment bound to calmodulin.. J Am Chem Soc.

[40] Herrmann T, Güntert P, Wüthrich K (2002). Protein NMR structure determination with automated NOE-identification in the NOESY spectra using the new software ATNOS.. J Biomol NMR.

[41] Herrmann T, Güntert P, Wüthrich K (2002). Protein NMR structure determination with automated NOE assignment using the new software CANDID and the torsion angle dynamics algorithm DYANA.. J Mol Biol.

[42] Zwahlen C, Legault P, Vincent SJF, Greenblatt J, Konrat R (1997). Methods for measurement of intermolecular NOEs by Multinuclear NMR spectroscopy: application to a bacteriophage λ N-peptide/*boxB* RNA complex.. J Am Chem Soc.

[43] Cornilescu G, Delaglio F, Bax A (1999). Protein backbone angle restraints from searching a database for chemical shift and sequence homology.. J Biomol NMR.

[44] Vuister GW, Bax A (1993). Quantitative J correlation: a new approach for measuring homonuclear three-bond J(HN-Hα) coupling constants in ^15^N-enriched proteins.. J Am Chem Soc.

[45] Güntert P (2004). Automated NMR structured calculation using CYANA.. Methods Mol Biol.

[46] Brunger AT, Adams PD, Clore GM, DeLano WL, Gros P (1998). Crystallography & NMR System (CNS). A new software suite for macromolecular structure determination.. Acta Crystallogr D.

[47] Nederveen AJ, Doreleijers JF, Vranken W, Miller Z, Spronk CA (2005). RECOORD: a REcalculated COORdinates Database of 500+ proteins from the PDB using restraints from the BioMagResBank.. Proteins.

[48] Ulyanov NB, Schmitz U, James TL (1993). Metropolis Monte Carlo calculations of DNA structure using internal coordinates and NMR distance restraints: An alternative method for generating a high-resolution solution structure.. J Biomol NMR.

[49] Wang J, Cieplak P, Kollman PA (2000). How well does a restrained electrostatic potential (RESP) model perform in calculating conformational energies of organic and biological molecules?. J Comput Chem.

[50] Dominguez C, Boelens R, Bonvin AM (2003). HADDOCK: a protein-protein docking aproach based on biochemical or biophysical information.. J Am Chem Soc.

[51] Zheng G, Lu XJ, Olson WK (2009). Web 3DNA-a web server for the analysis, reconstruction, and visualization of three-dimensional nucleic-acid structures.. Nucleic Acids Res.

[52] Rocchia W, Sridharan S, Nicholls A, Alexov E, Chiabrera A (2002). Rapid grid-based construction of the molecular surface and the use of induced surface charge to calculate reaction field energies: applications to the molecular systems and geometric objects.. J Comput Chem.

[53] Roessle MW, Klaering R, Ristau U, Robrahn B, Jahn D (2007). Upgrade of the small-angle X-ray scattering beamline X33 at the European Molecular Biology Laboratory, Hamburg.. J Appl Crystallogr.

[54] Konarev PV, Volkov VV, Sokolova AV, Koch MHJ, Svergun DI (2003). PRIMUS: a Windows PC-based system for small-angle scattering data analysis.. J Appl Crystallogr.

[55] Svergun DI, Barberato C, Koch MHJ (1995). CRYSOL-a program to evaluate X-ray solution scattering of biological macromolecules from atomic coordinates.. J Appl Crystallogr.

[56] Blackburn GM, Gait MJ, Loakes D, Willians DM (2006). Nucleic Acids in Chemistry and Biology..

[57] Laskowski RA, MacArthur MW, Moss DS, Thornton JM (1993). PROCHECK – a program to check the stereochemical quality of protein structures.. J Appl Crystallogr.

