# Supplementary figures and images for: Indirect DNA Readout by an H-NS Related Protein: Structure of the DNA Complex of the C-Terminal Domain of Ler
Source: PLoS Pathog. 2011 Nov 17;7(11):e1002380. doi: 10.1371/journal.ppat.1002380 (PMC3219716; doi:10.1371/journal.ppat.1002380)

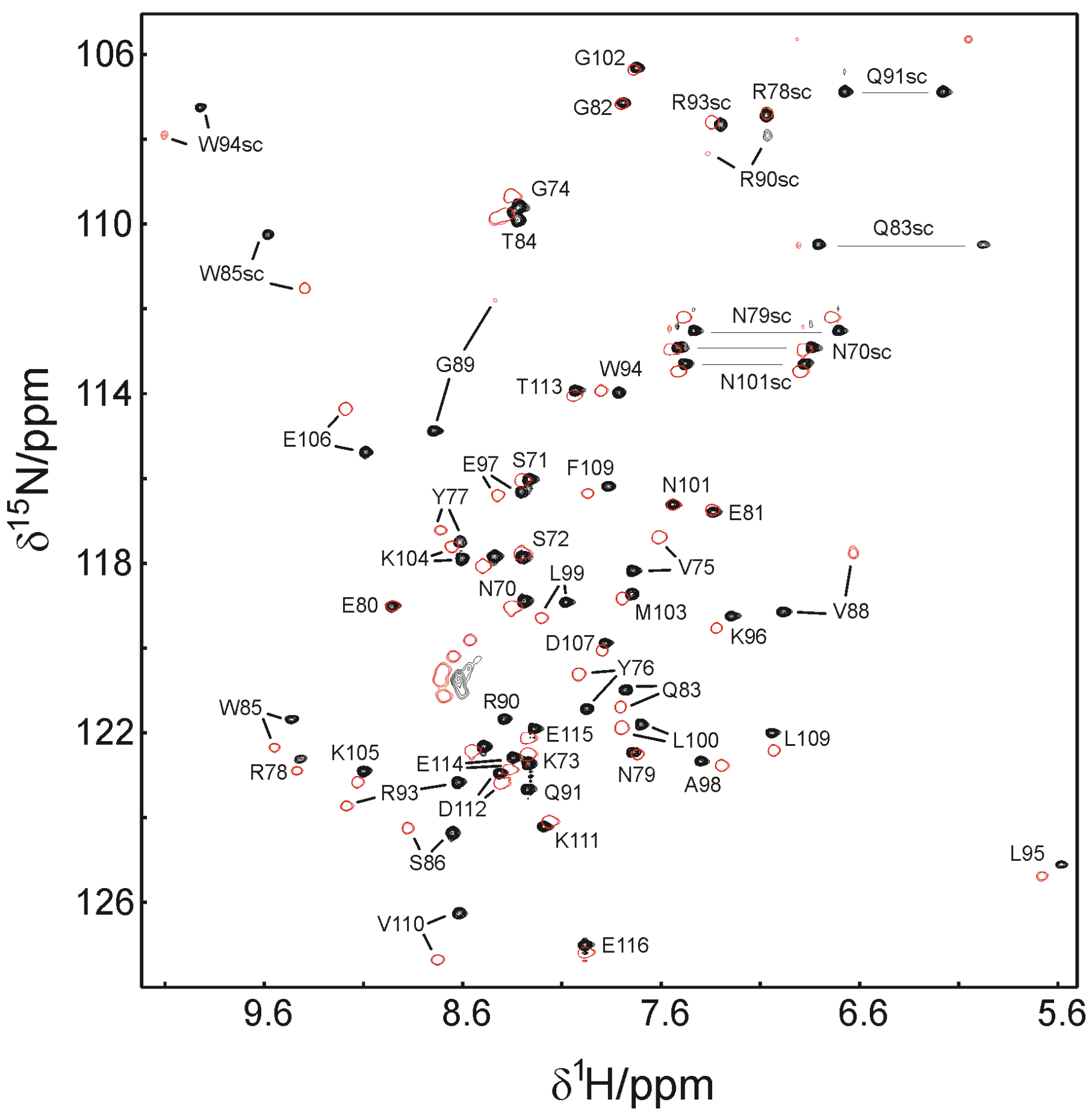

Supplement: Figure S1 — Interaction between CT-Ler and the LeeH dsDNA fragment. 1H-15N-HSQC NMR spectra of Ler70–116 (CT-Ler) recorded in the absence (black contours) and upon equimolar addition of LeeH (red contours). Side-chain NH groups are indicated by ‘sc’ after the residue number. The excellent chemical shift dispersion observed in the 1H-15N-HSQC NMR spectra indicates that the domain is properly folded. (TIF) [file ppat.1002380.s001.tif]

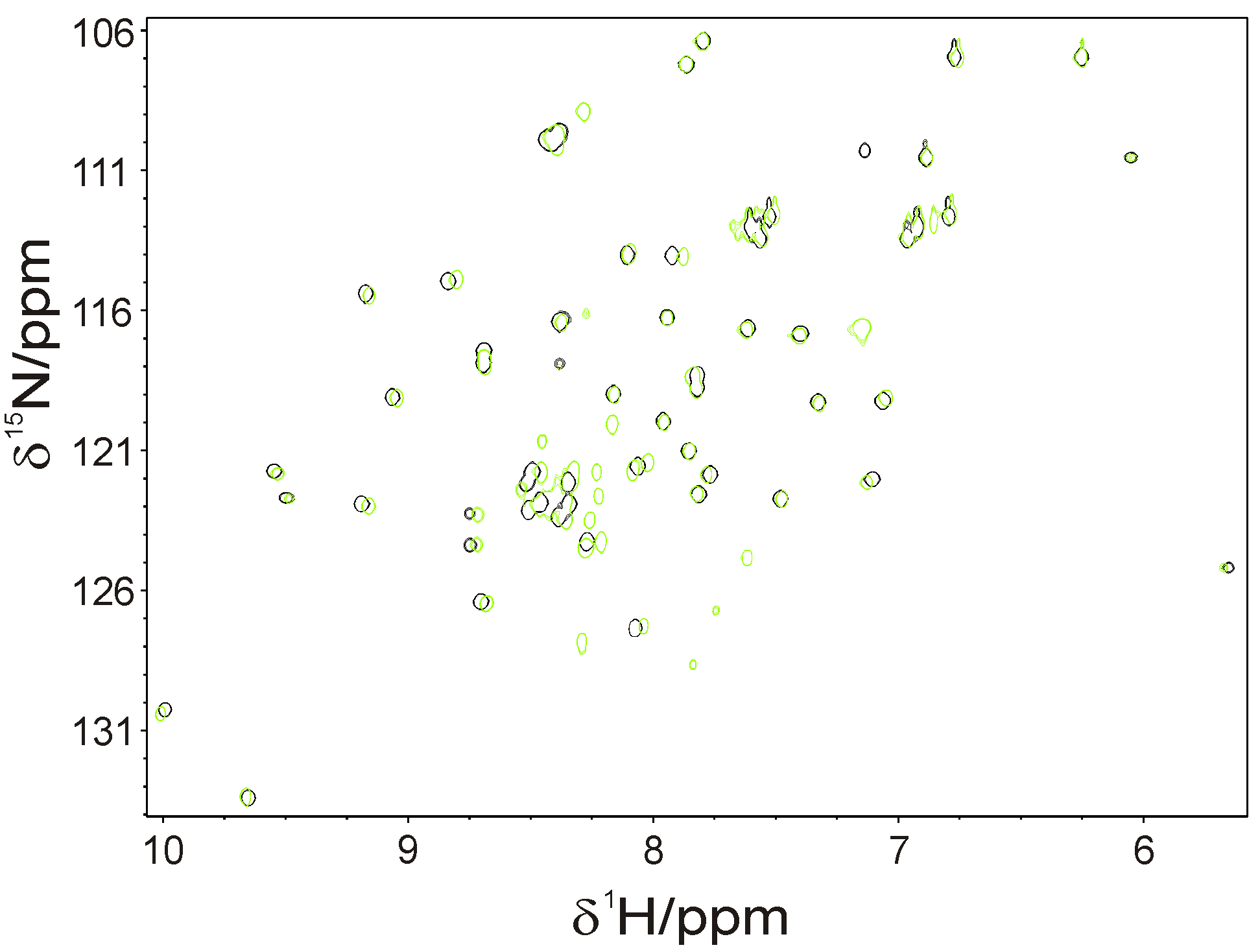

Supplement: Figure S2 — 1H-15N-HSQC spectra of Ler fragments. Overlap of 1H-15N-HSQC NMR spectra of Ler65–123 (green contours) and Ler70–116 (CT-Ler) (black contours) at pH 7.0 and 25°C. Most of the cross-peaks from CT-Ler coincide exactly with a cross-peak from Ler65–123. Additional residues from Ler65–123 show chemical shifts typical of unstructured residues. (TIF) [file ppat.1002380.s002.tif]

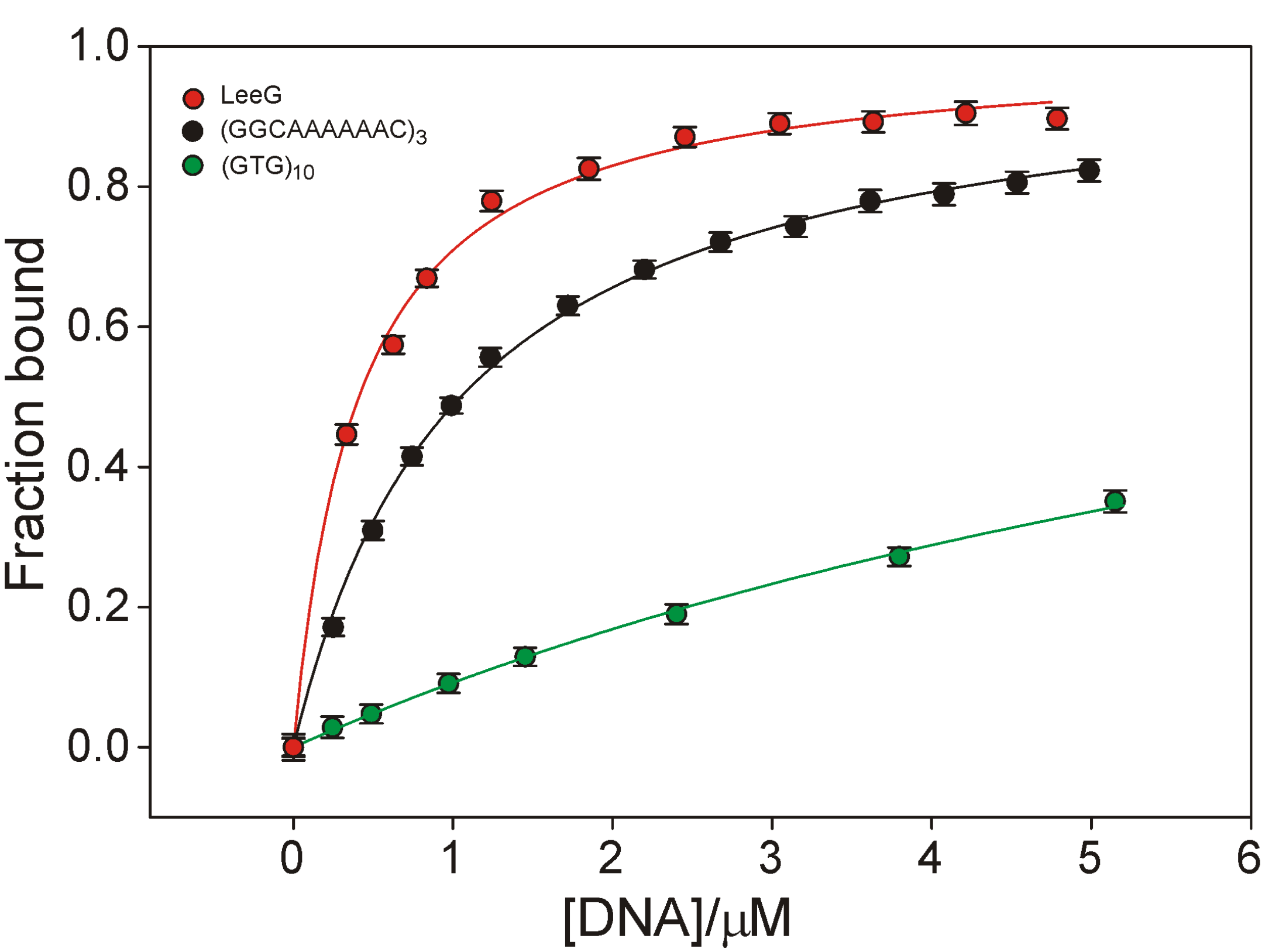

Supplement: Figure S3 — CT-Ler binds preferentially to AT-rich DNA sequences. Fluorescence anisotropy titrations of CT-Ler with the following 30-mer duplexes: LeeG, (GGCAAAAAAC)3 and (GTG)10. (TIF) [file ppat.1002380.s003.tif]

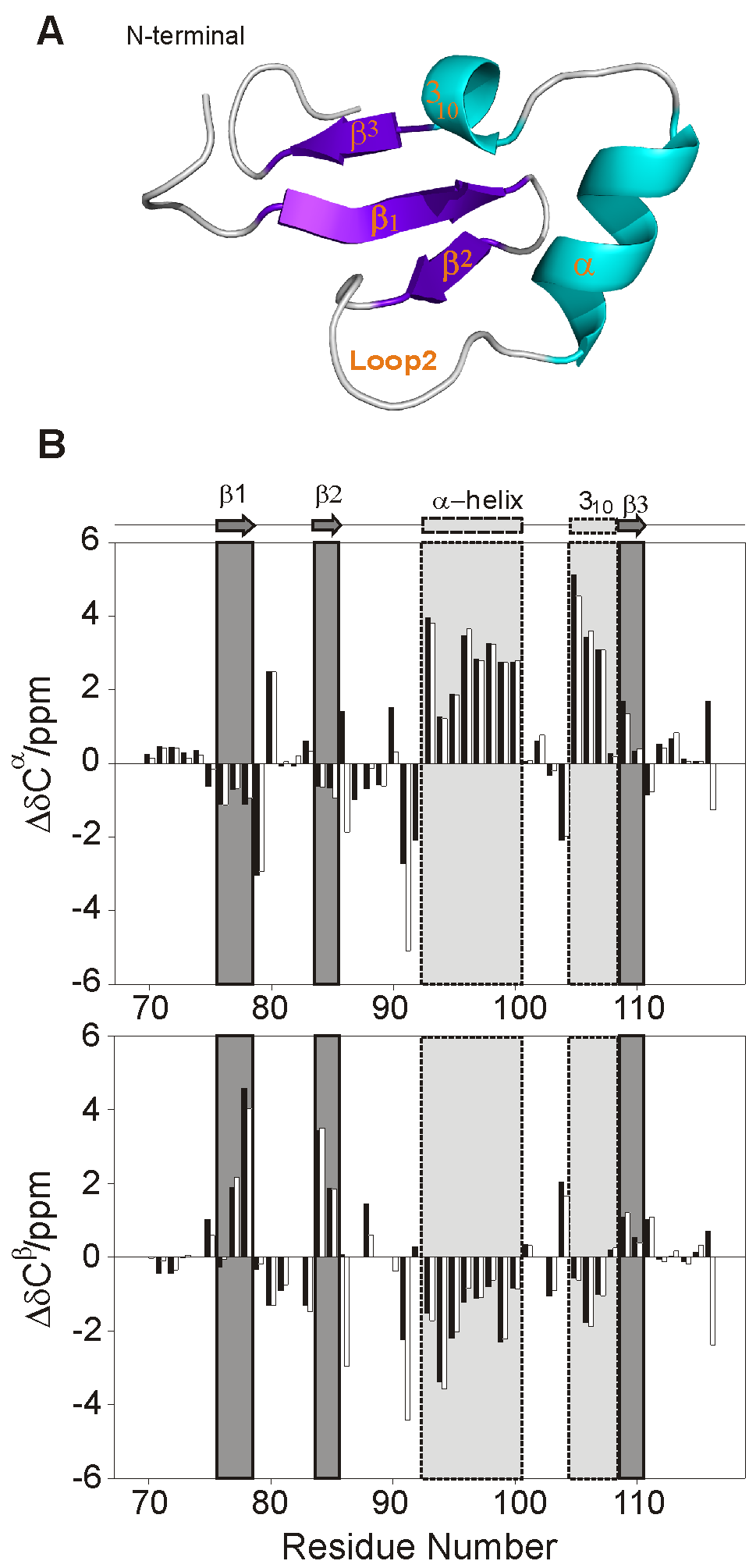

Supplement: Figure S5 — The CT-Ler secondary structure is not affected by DNA binding. (A) Ribbon structure of a representative conformer of LeeH-bound CT-Ler. Elements of secondary structure are labeled on the structure. (B) Differences between the 13Cα (top panel) and 13Cβ (bottom panel) chemical shifts observed for residues 70–116 of Ler and those expected for a random coil are plotted against the residue number. White and black bars correspond to the free and LeeH-bound forms, respectively. (TIF) [file ppat.1002380.s005.tif]
