# Supplementary material for: Indirect DNA Readout by an H-NS Related Protein: Structure of the DNA Complex of the C-Terminal Domain of Ler
Source: PLoS Pathog. 2011 Nov 17;7(11):e1002380. doi: 10.1371/journal.ppat.1002380 (PMC3219716; doi:10.1371/journal.ppat.1002380)
Supplement: Table S1 — DNA fragments used in the initial optimization of the CT-Ler/DNA complex. DNA fragments span the Ler-footprint within the LEE2/LEE3 regulatory region. Only the sequence of one of the complementary strands is shown. (DOC) [file ppat.1002380.s006.doc]

**Table S1 DNA fragments of the Ler-footprint within the *LEE2*/*LEE3* regulatory region.**

| **Fragment** | **Sequence (5’- 3’)^a^** |
| --- | --- |
| **LeeA** | TTGAAGAGTTTTTAAGATTGTTGGGAAATG |
| **LeeB** | GATTGTTGGGAAATGATTTTTATTTATTTG |
| **LeeC** | ATTTTTATTTATTTGATAACCGTGTTGAAA |
| **LeeD** | ATAACCGTGTTGAAATTGATTTTAATGGGT |
| **LeeE** | TTGATTTTAATGGGTTTTCTTTTTTTATTG |
| **LeeF** | TTTCTTTTTTTATTGAAATAATTGATAATA |
| **LeeG** | AAATAATTGATAATAATGTTTTTGTTACGT |

**^a^** Only the sequence of one of the complementary strands is shown
