# Supplementary material for: Indirect DNA Readout by an H-NS Related Protein: Structure of the DNA Complex of the C-Terminal Domain of Ler
Source: PLoS Pathog. 2011 Nov 17;7(11):e1002380. doi: 10.1371/journal.ppat.1002380 (PMC3219716; doi:10.1371/journal.ppat.1002380)
Supplement: Table S2 — NMR and refinement statistics. Refinement statistics including the number and type of experimental restraints and the results of quality controls performed using PROCHECK [57] and CRYSOL [55]. (DOC) [file ppat.1002380.s007.doc]

**Table S2 NMR and refinement statistics.**

|  | **CT-Ler**^a^ |
| --- | --- |
| **NMR distance and dihedral constraints** |  |
| *NOE restraints* | 1302 |
| Intra-residue | 244 |
| Inter-residue | 1058 |
| Sequential (|*i* – *j*| = 1) | 375 |
| Nonsequential (|*i* – *j*| > 1 ) | 683 |
| Medium range (1<|*i* – *j*| ≤ 4 ) | 378 |
| Long range (|*i* – *j*| > 4 ) | 305 |
| *Hydrogen bonds*^b^ | 12 |
| *Dihedral angle restraints* | 96 |
| φ | 35 |
| ψ | 29 |
| χ_1_ | 32 |
| **Structure statistics** |  |
| *Violations* |  |
| Distance constraints (Å) | 0.024±0.004 |
| Dihedral angle constraints (º) | 0.53±0.53 |
| *Deviations from idealized geometry* |  |
| Bond lengths (Å) | 0.0038±0.0077 |
| Bond angles (º) | 0.14±0.37 |
| *Average pairwise RMSD^c^* (Å) |  |
| Heavy atoms | 1.48±0.20 |
| Backbone atoms | 0.93±0.18 |
| **Ramachandran Analysis**^d^ |  |
| Most favoured regions | 85.52±4.57 % |
| Additional allowed regions | 12.18±4.06 % |
| Generously allowed regions | 2.05±1.58 % |
| Disallowed regions^e^ | 0.26±0.79 % |
|  | **Complex** |
| ***i*restraints** |  |
| *iNOE restraints* | 30 |
| *AIRS* | 12 |
|  |  |
| **Structure statistics** |  |
| *Violations* |  |
| *i*restraints deviations | 0.37±0.12 |
| *Deviations from idealized geometry* |  |
| Bond lengths (Å) | 0.0030±0.0001 |
| Bond angles (º) | 0.50±0.03 |
| *Average pairwise RMSD^c^* (Å) |  |
| Heavy atoms | 1.30±0.38 |
|  |  |
| **Ramachandran Analysis**^d^ |  |
| Most favoured regions | 86.15±3.57% |
| Additional allowed regions | 12.10±2.69% |
| Generously allowed regions | 1.15±1.94% |
| Disallowed regions | 0% |
|  |  |
| **CRYSOL fitting statistics** |  |
| $\chi_{Ensemble}$^f^ | 1.24±0.04 |

^a^ Experimental restraints were obtained in the presence of DNA. ^b^ Hydrogen bonds were identified on the basis of strong protection factors. Two restraints per hydrogen bond were included in the calculation (H_N_−O ≤ 2.0Å and N−O ≤ 3.0Å). ^c^ Pairwise RMSD was calculated among the 20 refined structures. ^d^ Ramachandran analysis was performed using PROCHECK. Only CT-Ler residues were included in the analysis. ^e^ R90 was in disallowed regions in two out of twenty conformers when DNA was omitted in the calculation. ^f^$\left\langle\chi_{Ensemble}\right\rangle$value defines the goodness of the CRYSOL fitting to SAXS data.
