# Supplementary material for: Indirect DNA Readout by an H-NS Related Protein: Structure of the DNA Complex of the C-Terminal Domain of Ler
Source: PLoS Pathog. 2011 Nov 17;7(11):e1002380. doi: 10.1371/journal.ppat.1002380 (PMC3219716; doi:10.1371/journal.ppat.1002380)
Supplement: Table S3 — DNA sequence effect on CT-Ler complex stability. Sequences of the LeeH variants designed to test CT-Ler binding specificity and the corresponding dissociation constants. Only the sequence of one of the complementary strands is shown. (DOC) [file ppat.1002380.s008.doc]

**Table S3 DNA sequence effect on CT-Ler complex stability^a^.**

| **LeeH variants** | **Sequence (5’- 3’)** | ***K_d_ /μM*** |
| --- | --- | --- |
| WT | GCGATAATTGATACC | 1.10±0.05 |
| A4G | GCG**G**TAATTGATACC | 1.06±0.08 |
| T5C | GCGA**C**AATTGATACC | 1.38±0.11 |
| A6G | GCGAT**G**ATTGATACC | 2.48±0.20 |
| A7G | GCGATA**G**TTGATACC | 2.71±0.32 |
| T8C | GCGATAA**C**TGATACC | 1.70±0.13 |
| T9C | GCGATAAT**C**GATACC | 1.36±0.08 |
| G10A | GCGATAATT**A**ATACC | 2.31±0.07 |
| A11G | GCGATAATTG**G**TACC | 1.43±0.04 |
| T12C | GCGATAATTGA**C**ACC | 1.45±0.10 |
| A13G | GCGATAATTGAT**G**CC | 1.48±0.09 |
| **LeeH core variants** | **Sequence (5’- 3’)** | ***K_d_ /μM*** |
| AATT | CGCAATTGCGH | 3.45±0.56 |
| AAAT | CGCAA**A**TGCGH | 6.17±0.36 |
| AATA | CGCAAT**A**GCGH | 11.46±0.96 |
| AATTC | CG**G**AATT**C**CGH | 4.14±0.31 |
| AAAA | CGCAA**AA**GCGH | 8.07±0.22 |
| TATA | CGC**T**AT**A**GCGH | 16.57±2.20 |
| TTAA | CGC**TTAA**GCGH | 22.83±2.99 |

**^a^** Only the sequence of one of the complementary strands is shown

Nucleotide substitutions are shown in bold.
